# On the feasibility of accessing acute pain–related facial expressions in the human fetus and its potential implications: a case report

**DOI:** 10.1097/PR9.0000000000000673

**Published:** 2018-07-31

**Authors:** Lisandra Stein Bernardes, Juliana Fontan Ottolia, Marina Cecchini, Antônio Gomes de Amorim Filho, Manoel Jacobsen Teixeira, Rossana Pulcineli Vieira Francisco, Daniel Ciampi de Andrade, Grupo de Estudo da Dor Fetal (Fetal Pain Study Group)

**Affiliations:** a Disciplina de Obstetrícia, Departamento de Obstetrícia e Ginecologia, Faculdade de Medicina FMUSP, Universidade de São Paulo, São Paulo, Brazil; b Pain Center, Department of Neurology, University of São Paulo, São Paulo, Brazil; c Pain Center, Instituto do Câncer do Estado de São Paulo Octavio Frias de Oliveira, University of São Paulo, São Paulo, Brazil

**Keywords:** Fetal pain, Experimental pain, Acute pain, Prenatal, Fetal surgery, Pregnancy

## Abstract

**Introduction:**

Although pain facial assessment is routinely performed in term and preterm newborns by the use of facial expression–based tools such as the Neonatal Facial Coding System, the assessment of pain during the intrauterine life has not been extensively explored.

**Objective:**

Describe for the first time, an experimental model to assess and quantify responses due to acute pain in fetuses undergoing anaesthesia for intrauterine surgery recorded by high-resolution 4D ultrasound machines.

**Methods/results-case report:**

A 33-year-old pregnant woman had congenital left diaphragmatic hernia of poor prognosis diagnosed, and her fetus was treated by fetoscopic endotracheal occlusion. Later, during the removal of the fetal endotracheal balloon by ultrasound-guided puncture, we have recorded facial expressions of the foetus before and after the anaesthetic puncture by the use of 4D ultrasound recordings, which were presented to 3 blinded coders instructed to use the Neonatal Facial Coding System for acute pain facial coding. The procedure was safe and feasible.

**Conclusion:**

This is the first description of a recordable acute pain model in the human fetus by the use of a facial expression–based tool. The possibility to assess pain-related intrauterine behaviours would allow not only for the monitoring of the efficacy of anaesthetic procedures in the fetus but would also open the way to explore the evolution of pain-related facial responses during the fetal neurodevelopment. This method may pave the way for objective assessments of pain in fetuses, should it endure the steps of formal validation studies.

## Abstract 2

Supplemental Digital Content is Available in the Text.

## 1. Introduction

The assessment of the presence of pain during the intrauterine life has important practical and theoretical consequences. It has been argued that from the 20th gestational week onwards, fetuses have brain structures anatomically developed potentially allowing for salient nociceptive inputs to trigger withdraw reflexes away from inciting nociceptive stimuli, which in turn, could also be associated with the experience of pain.^13,15,16,24^ However, studies discussing foetal pain had been inconsistent, and no model for the evaluation of foetal pain had been described until now.^4,20,23^ From a pragmatic clinical perspective, the presence of pain in foetuses and the possibility to assess it would require a series of additional actions from health care providers dedicated to fetal medicine. One could suggest that anaesthesia should be systematically provided not only to fetuses undergoing intrauterine procedures or surgeries but also to those who will undergo medical abortion after the 20th gestational week.^1,6,26^ And, this discussion could further branch off into the issue of monitoring the efficacy of intrauterine analgesia during invasive intrauterine procedures and even monitor the presence of chronic pain in fetuses undergoing potentially painful chronic procedures, such as intrauterine correction of neural tube defects. None of these challenges have easy solutions. Interestingly, pain physicians are well used to assess pain and pain behaviours in humans in instances where verbal communication does not exist or is impaired, such as newborns, young children, language-impaired, or adults with advanced dementia.^3,17,19^ Pain assessment in these settings is highly dependent on facial expressions, one of the hallmarks of painful behaviours in humans and in several mammals.^12,14,18^ In fact, easy-to-perform pain behavioural assessments are routinely performed in term and preterm newborns worldwide by the use of facial expression–based scales such as the Neonatal Facial Coding System (NFCS).^9^ In the past years, with the development of high-resolution 4D ultrasound machines, fetal medicine specialists have gained access to high-resolution images of the fetus, which has allowed for the performance of intrauterine procedures with high efficacy rates and better outcomes.^2,7^ In the past years, several centers have reported on the facial expressions of fetuses in different conditions during the intrauterine development by using this imaging technology.^28^ In one previous study, it has actually been tried to use a standardized facial scoring system–based tool (Facial Action Coding System)^8^ to assess the presence of potential pain-related facial expressions in normal fetuses, which was an original and innovative approach.^25^ However, this report was uncontrolled, and fetuses were simply and passively observed in the absence of any type of stimulation, which put into doubt whether the different facial expressions reported were simply physiological facial movements seen during the normal human development, or were anyhow related to some (unseen) painful experience. Moreover, facial movement combinations supposed to be related to “pain gestalt” were defined by the authors and not validated based on an actual fetal painful situation. Here, we described, for the first time, an experimental model of acute pain in fetuses undergoing anaesthesia for an intrauterine procedure. Because in this setting there is a clear noxious stimulation (ie, anaesthetic injection puncture), facial expressions collected before the intramuscular shot were compared with those recorded immediately after the procedure. We have reported that the use of the NFCS is feasible to detect pain-related facial expressions compared with the rest condition in a randomized and blinded assessment report. Once fully and formally validated, this tool may allow for the monitoring of analgesic treatment during fetal procedures and may pave the way to gain deeper insights into the possible presence of pain behaviours in fetuses with long-standing conditions such as gastroschisis, postmyelomeningocele, and restricted intrauterine growth.

## 2. Case report

**Figure 1.**
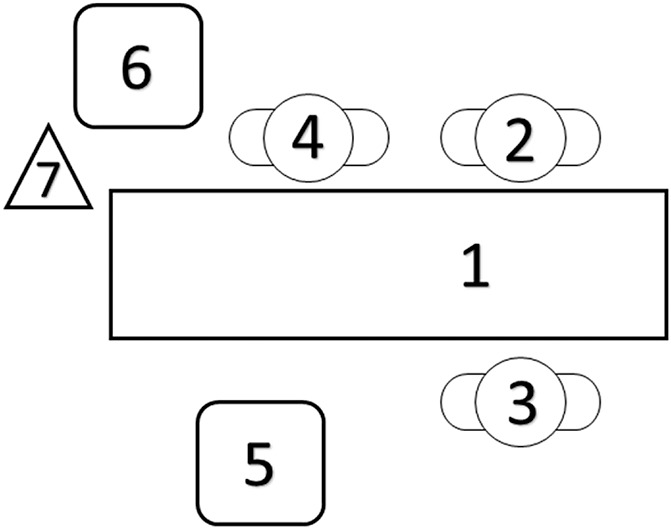
The spatial set-up of the fetal surgery and face recording. (1) Position of the mother; (2) chief surgeon—performed the puncture; (3) assistant surgeon—obtained the 4D images; (4) surgical technologist; (5) ultrasound machine (Voluson 730; GE Health-care) used in surgery focusing the fetal trachea/thigh; (6) the 4D ultrasound machine (Voluson E8; GE Health-care) used for fetal face recording; and (7) an external camera (iSight camera, 8-megapixel with 1.5 µ pixels, autofocus; f/2.2 aperture, optical image stabilization, 1080p, 60 FPS HD video recording from iPhone 6 [Apple, California] held in a tripod. Images were edited in iMovie [Apple, California] offline using 100% shake reduction and no other filter or image edition except for the duration of the recording).

A 33-year-old pregnant woman (3 gestations, 1 delivery, and 1 abortion) had antenatal diagnosis of gestational diabetes mellitus, which was treated by dietary control. A preplanned ultrasound assessment detected the presence of congenital left diaphragmatic hernia of poor prognosis (initial lung area to head circumference ratio = 0.85) and normal fetal karyotype. A fetoscopic endotracheal occlusion was successfully performed at 28 weeks 1/7 days gestation with subsequent improvement of lung area to head circumference ratio to 1.07. During a follow-up ultrasound examination performed at 32 gestational weeks, the mother complained of lower belly pain and contractions. On physical examination, she presented 4 uterine contractions in 30 minutes and had cervicodilatation of 2 cm. She was then hospitalized to remove the fetal endotracheal balloon by ultrasound-guided puncture to prevent fetal asphyxia after delivery and placental detachment. Before the procedure, the fetus was routinely anesthetized with an intramuscular injection on the left thigh (targeting the quadriceps muscle) containing pancuronium (0.50 mg/0.25 mL) and fentanyl (40.0 μg/0.8 mL), using a 20 G × 6 in. needle. After written informed consent was provided by the mother, and for the purposes of assessing putative pain-related behaviours noninvasively during the procedure, we have added a second ultrasound machine in the operating room (Voluson E8; GE Health-care, Zipf, Austria) operated by a second foetal medicine specialist exclusively to monitor the facial expressions of the foetus during the anaesthetic puncture. We have recorded the preanaesthetic and postanaesthetic 4D ultrasound films and presented it to 3 coders to assess facial expressions using the NFCS, which is validated to detect pain behaviours and suffering healthy and preterm newborns, but never before used during the intrauterine life during acute pain conditions (Fig. 1 and Supplemental Digital Content, http://links.lww.com/PR9/A27).^10^ The 10 facial actions of the NFCS were coded: brow lowering, eyes squeezed shut, deepening of the nasolabial furrow, open lips, vertical mouth stretch, horizontal mouth stretch, taut tongue (cupping of the tongue), chin quiver (high frequency vibration of the chin), lip purse (tightening the muscles around the lips to form “oo”), and tongue protrusion. Each face action was classified as visible or not, and, if visible, they were coded as 1/0 (occurred/did not occur). Four dimensional ultrasound images were recorded before and after the anaesthetic procedure and were anonymized for off-line assessment including: (1) a baseline period defined as the least 30 seconds before the anaesthesia puncture and (2) the 45 seconds immediately after the puncture (Supplemental Digital Content, http://links.lww.com/PR9/A27). Thus, video extracts were presented to 3 coders with no previous background in behavioural coding: a neurologist, a psychologist, and a fetal medicine obstetrician specialized in 4D ultrasound of foetuses. All coders were blinded to the timing of the videos (before vs after puncture), which were randomly presented to each coder (scores were illustrated in Tables 1).

**Table 1:**
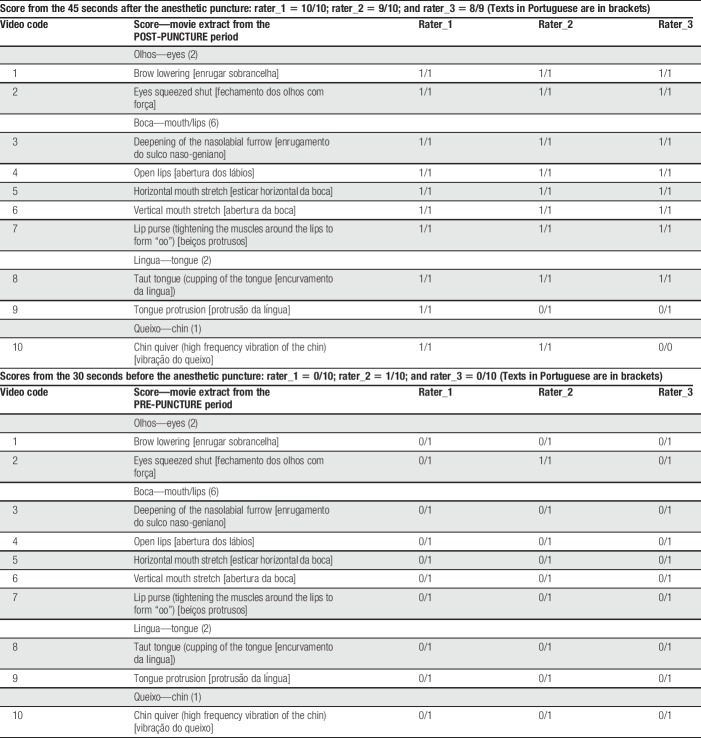
Results obtained from the video scoring using NFCS

## 3. Discussion

This is the first attempt, to the best of our knowledge, to describe the feasibility of using a facial expression–based tool, which was originally developed for newborns, in a human fetus under an acute pain condition. We have described the fetal facial changes seen after acute pain by using high-resolution 4D ultrasound. In this model, we have used the rest condition before the injection of analgesics as a control, to explore the facial expressions that were present exclusively during the painful anaesthetic puncture. Should this approach be confirmed in future larger validation studies, it could be a way to monitor acute pain–related facial expression in human fetuses in an inexpensive and straightforward fashion. Indeed, the possibility to assess pain-related behaviours *intrauterus* would allow not only for the monitoring of the efficacy of anaesthetic procedures in the fetus but would also open the way to explore the evolution of pain-related facial responses during the fetal neurodevelopment. One could explore when the pain-related facial expressions actually begin during the development, and which are their core components.^25^ It could also open the way to the development of a potential biological markers of pain/pain behaviours in fetus,^22^ which can have ethical and legal consequences. However, no human studies have directly reported on the development of thalamocortical circuits associated with pain perception in fetuses.^20^ From the theoretical point of view, one frequently associates the presence and qualities of facial expressions in pain with the social-adaptative development of nonverbal communication.^11,27^ Since fetuses express a plethora of facial movements in the dark uterus, one could ask whether this energy-consuming and apparently socially vital behaviour is being trained for extrauterine life (such as other behaviours such as sucking and stretching), or would it be serving another function. The presence of such facial movements would actually favour the model,^5^ proposing that feelings are indeed a coupling of the sensory discriminant perception with an “output” behavioural drive and reflex response. In this view, facial expressions may constitute the motor reflexive expression of the emotional motor system and could exist in the absence of a socially interacting observer.^21^ The aim of this report was to provide the description of an acute pain model and a potentially useful way to measure it. Should this approach endure the processes of validation, it could bring to life practical as well as theoretical new insights into the mechanisms of pain expression in humans.

## Disclosures

There are no conflicts of interests in this study.

This study was funded by the Pain Center and Department of Obstetrics and Gynecology.

## Appendix A. Supplemental digital content

Supplemental digital content associated with this article can be found online at http://links.lww.com/PR9/A27.
